# Use of a specific antidote to dabigatran (idarucizumab) reduces blood loss and mortality in dabigatran-induced and trauma-induced bleeding in pigs

**DOI:** 10.1186/cc13289

**Published:** 2014-03-17

**Authors:** M Honickel, O Grottke, J Van Ryn, H Ten Cate, R Rossaint, H Spronk

**Affiliations:** 1 RWTH Aachen, Germany; 2 Boehringer Ingelheim, Biberach, Germany; 3 Maastricht University, Maastricht, the Netherlands

## Introduction

The reversal of the anticoagulant effects of the new oral anticoagulants in severely bleeding patients requires new therapeutic strategies. This study investigated the effectiveness of a specific antidote for idarucizumab to reverse bleeding in a dabigatran anticoagulated pig trauma model.

## Methods

After ethical approval, male pigs (*n *= 30) were given dabigatran etexilate for 3 days (30 mg/kg bid p.o.), and the sham group (*n *= 6) received placebo. To achieve supra-therapeutic anticoagulation, dabigatran was infused prior to injury on day 4 in anesthetized pigs, and the sham group received placebo. A standardized blunt liver injury was inflicted and blood loss (BL) was recorded 10 minutes post trauma. The dabigatran-treated animals were randomized (*n *= 6/group) to a single injection of idarucizumab at 30, 60 or 120 mg/kg i.v. or vehicle (control animals). Blood loss and hemodynamic variables were monitored over 4 hours or until time of death. Data were analyzed by ANOVA (± SD) and by the log-rank test.

## Results

Dabigatran levels were 1,147 ± 370 ng/ml with no differences between groups prior to injury. BL in sham animals was 409 ± 53 ml 10 minutes after injury and 700 ± 107 ml after 4 hours (survival rate 100%). Anticoagulation with dabigatran (control animals) resulted in significantly higher BL 10 minutes after injury (801 ± 66 ml, *P *< 0.05). Mortality in these animals was 100%, with a mean survival time of 121 minutes (range: 90 to 153 minutes; *P *< 0.05 vs. sham and idarucizumab-treated animals). Total BL in dabigatran-treated animals was 2,977 ± 316 ml. In contrast, treatment with idarucizumab was associated with a dose-dependent reduction in BL. The lowest dose of 30 mg/kg resulted in 17% mortality (1/6 animals) and BL was reduced by 50% (1,586 ± 619 ml). BL was further reduced in animals receiving 60 (1,077 ± 103 ml) or 120 mg/kg idarucizumab (1,137 ± 121 ml; both 100% survival). Hemodynamic parameters and markers of shock were similar to pre-trauma levels in latter groups receiving idarucizumab.

## Conclusion

This study demonstrates for the first time that anticoagulation with dabigatran can be reversed effectively and safely by idarucizumab. Even under supra-therapeutic dabigatran concentrations, the antidote decreased blood loss and mortality in this lethal pig animal model. Thus, data from clinical studies are warranted to confirm the findings of this study.

